# Comparative transcriptome analysis of two reproductive modes in *Adiantum reniforme* var. *sinense* targeted to explore possible mechanism of apogamy

**DOI:** 10.1186/s12863-019-0762-8

**Published:** 2019-07-09

**Authors:** Qi Fu, Long-qing Chen

**Affiliations:** 1 Key Laboratory of Horticultural Plant Biology, Ministry of Education/College of Horticulture and Forestry Sciences, Huazhong Agricultural University, Kunming, 6502240 China; 2 Southwest Research Center of Landscape Architecture Engineering (State Forestry and Grassland Administration), Southwest Forestry Universityy, Kunming, 650224 China

**Keywords:** Apogamy, Fern, *Adiantum reniforme* var. *sinense*, Gametophyte, Transcriptome

## Abstract

**Background:**

Apogamy is a unique asexual reproduction in the ferns, in which somatic cells of gametophytes go through dedifferentiation and then differentiate into haploid sporophytes bypassing fertilization. Restricted to the lack of genomic information, molecular mechanisms of apogamy have remained unclear. Comparative transcriptome analysis was conducted at six stages between sexual reproduction and apogamy in the fern *Adiantum reniforme* var. *sinense*, in an effort to identify genes and pathways that might initiate the asexual reproduction.

**Results:**

Approximately 928 million high-quality clean reads were assembled into 264,791 unigenes with an average length of 615 bp. A total of 147,865 (55.84%) unigenes were successfully annotated. Differential genes expression analysis indicated that transcriptional regulation was more active in the early stage of apogamy compared to sexual reproduction. Further comparative analysis of the enriched pathways between the early stages of the two reproductive modes demonstrated that starch and sucrose metabolism pathway responsible for cell wall was only significantly enriched in asexual embryonic cell initiation. Furthermore, regulation of plant hormone related genes was more vigorous in apogamy initiation.

**Conclusion:**

These findings would be useful for revealing the initiation of apogamy and further understanding of the mechanisms related to asexual reproduction.

**Electronic supplementary material:**

The online version of this article (10.1186/s12863-019-0762-8) contains supplementary material, which is available to authorized users.

## Background

As popular ornamental foliage plants, the ferns are now cultivated in many countries and have gradually gained more and more attention and increasing demand all over the world [1]. The species such as Adiantum and Drynaria are also considered as medicinal plants in curing the human disease in multiple fields. The central question for utilization of ferns concerns exploiting new germplasm resources and increasing the reproductive efficiency. Ferns are unique among land plants in having separate and autotrophic gametophytes and sporophytes. The gametophyte-to-sporophyte ways for ferns possess two reproductive modes: sexual reproduction and apogamy [2, 3]. Unlike the sexual reproduction features fertilization and zygotic embryogenesis, in apogamy the sporophytes are formed directly from somatic cells of gametophytes, without the intervention of sexual organs. It is estimated that 10% of the extant fern species reproduce obligately asexually by apogamy, perhaps as an adaptation to a harsh environment [4–6].

The induction of apogamy has been accomplished in many ferns by culturing gametophytes on exogenous substances, such as carbohydrates [2, 7], osmotic conditions [8], and plant hormones including ethylene [9], auxins [10] and cytokinins [11]. However, the molecular mechanism and related signaling networks in controlling apogamy is still not entirely understood. So far, there are several studies focusing on the genes promoting the apogamy in *Physcomitrella patens* [12] and *Ceratopteris richardii* [13, 14], trying to interpret the phenomenon. In *P. patens*, apogamy could result from deletion of the gene orthologous to the *Arabidopsis thaliana* CURLY LEAF (*CLF*), which encodes a component of Polycomb Repressive Complex 2 (PRC2). In *C. richardii*, overexpression of *CrANT* or *BnBBM* could promote apogamy. In addition, apogamy commitment was suggested to be associated with stress and metabolism by suppression subtractive hybridization in *C. richardii*. In the apogamous fern *Dryopteris affinis* ssp. *affinis*, the phytohormone signaling and stress responses were proposed to participate in vegetative and reproductive gametophyte development by comparative analysis between transcriptome and proteogenome [15].

In apogamy, the pivotal step is that somatic cells of the gametophyte are reprogrammed to start the sporophytic developmental program. It shows a remarkable phenomenon of cell totipotency, which is just like the feature of somatic embryogenesis in seed plants. Coincidentally, a *CrANT* gene in *Ceratopteris richardii*, mirroring BABY BOOM in *Brassica napus*, has been certified to promote apogamy, indicating the genetic similarity between apogamy in ferns and somatic embryogenesis in angiosperms [14]. Generally, it is believed that the genes responsible for cell division and cell wall, and the genes related to plant hormones play a key role in triggering vegetative-to-embryogenic transition [16–18]. The studies of genes and signaling pathways related to vegetative-to-embryogenic transition would be benefit for understanding the mechanism triggering apogamy. With the further comparative research of the key genes, the evolutionary process of asexual reproduction might gradually become clear.

*Adiantum reniforme* var. *sinense*, an unifoliate species that belongs to the family of Adiantaceae, is a rare and endangered fern distributed in China [19, 20]. The fern has been successfully induced in vitro to form sporophytes through sexual reproduction and apogamy respectively in the previous research. The morphological and cytological observations revealed that no sexual organs differentiated on the gametophytes during the process of apogamy. The sporophytes developed with more number and faster frequency on a single gametophyte in apogamy compared with sexual reproduction, reflecting higher reproductive efficiency. The progeny would inherit the trait based on the character of asexual reproduction. Comparative studies of key stages of the two reproductive modes would constitute a significant step forward in understanding apogamy initiation.

This study is aiming to uncover the possible molecular mechanisms of apogamy in ferns at transcriptional level. Due to *A. reniforme* var. *sinense* is lack of reference genome, de novo assembly using high-throughput sequencing technology is an ideal method for transcriptome analysis. Therefore, RNA-seq was performed to explore the gene expression differences in gametophytes at six stages between sexual reproduction and apogamy. Comparative transcriptome analysis of early stages between the two reproductive modes was carried out to clarify the genetic basis for apogamy initiation. To our knowledge, this is the first attempt to use RNA-Seq to detect differential expression of genes in fern gametophytes conducting two reproductive modes. On the basis of bioinformatics analysis, it is expected that the results will help to reveal molecular mechanisms that responsible for apogamy in the ferns. In the future, the research on the function of key genes will be beneficial to create new germplasms with high reproductive efficiency in other ferns by transgenic technology.

## Results

### The morphogenetic characteristics of gametophytes for two reproductive modes

**Fig. 1:**
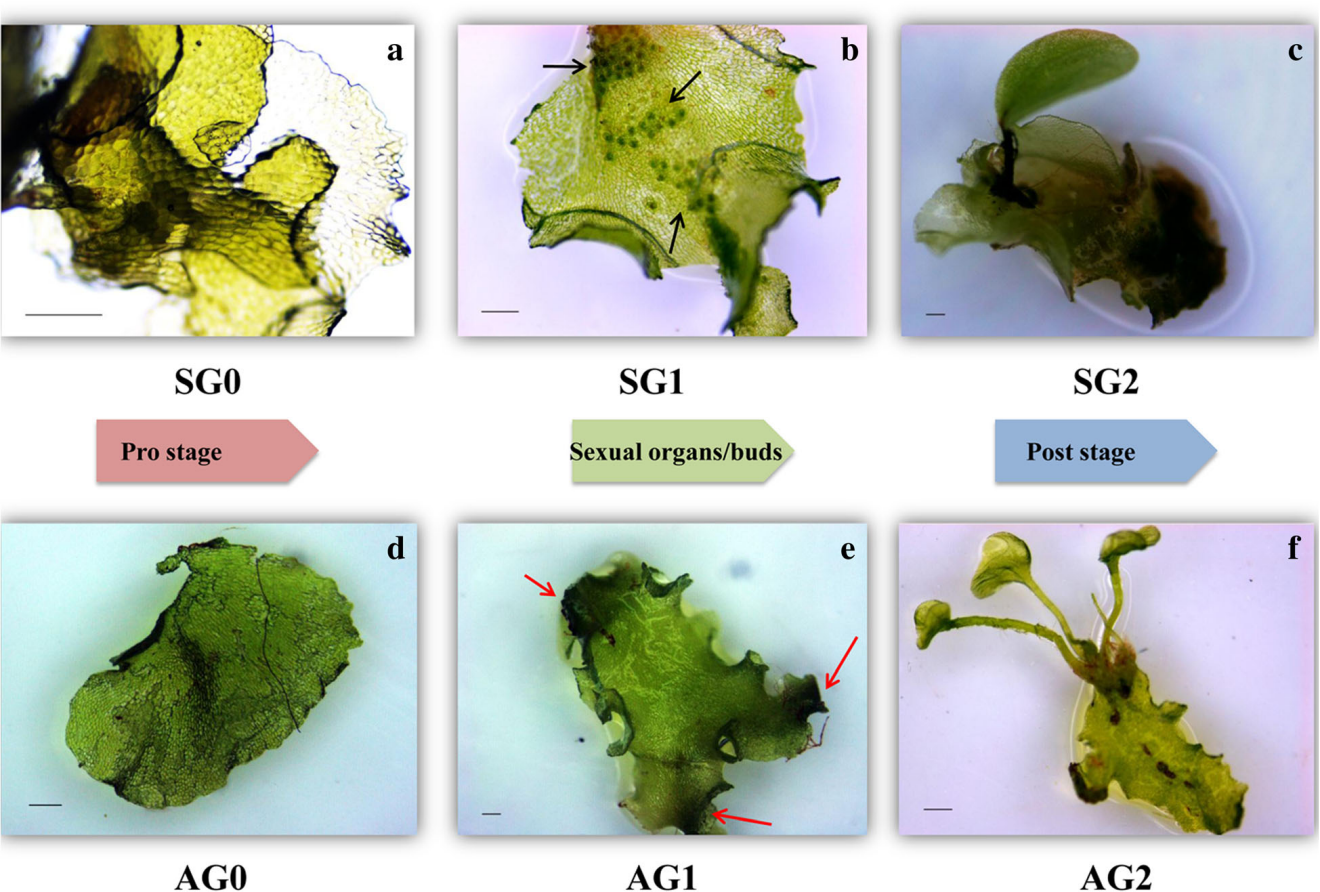
Sexual reproduction and apogamy development stages in *A. reniforme* var. *sinense* gametophytes. **a** gametophyte with no sexual organs differentiation (SG0); **b** gametophyte with sexual organs initiation (SG1); **c** gametophyte with the sporophytes appeared (SG2); **d** gametophyte with no agamous buds appearing (AG0); **e** gametophyte with agamous embryonic cells initiation (AG1); **f** gametophyte with the apogamous sporophytes appeared (AG2). Black arrows, sexual organs; red arrows, asexual embryonic cells; Bar = 1 mm

The reproductive status was defined by the morphologies of the gametophytes before construction of transcriptome library. In Fig. 1, the SG0, SG1 and SG2 respectively represented as the early, middle and later stages of sexual organs differentiation in sexual reproduction. Correspondingly, the AG0, AG1 and AG2 represented as the early, middle and later stages of asexual embryonic cells in apogamy. The gametophytes were morphologically similar between SG0 and AG0. The color was bright green and the thickness was slight. No sexual organs or asexual embryonic cells emerged on the surface. At SG1 stage, as the critical period of sexual organ differentiation, many outgrowths (namely sexual organs, including archegonia and antheridia) appeared on the gametophytes. The archegonia and antheridia were generally located in the middle and edge of gametophytes respectively. The color of sexual organs was green and darker than surrounding cells obviously. At AG1 stage, at the edge of gametophytes, somatic cells transformed to asexual embryonic cells and proliferated continually. The color was dark green obviously at these sites. These cells were extended to be the form of buds and then developed to sporophytes later. The two kinds of gametophytes presented different reproductive characteristics. The gametophytes both developed sporophytes at the stages of SG2 and AG2. The difference was that the apogamous gametophytes produced more sporophytes than sexual reproductive gametophytes. In addition, the juvenile fronds of sporophytes were oval shape in sexual reproduction and reniform shape in apogamy.

### De novo assembly of gametophytes transcriptome

The high-throughput Illumina strand-specific RNA sequencing technology was employed to sequence the six stages of two reproductive patterns (three biological replicates for each stage, two biological replicates for SG2). Each sample was represented by at least 25 million reads. In total, 980 million short reads were generated from all samples, with 928 million high-quality clean reads selected for further analysis. The de novo assembly was conducted by using Trinity software according to standard parameters. The assembly resulted in a total of 333,352 transcripts (no less than 200 bp) with a N50 of 1555 bp, and 264,791 unigenes with a N50 of 951 bp. The column diagrams of size distribution of transcripts and unigenes were shown (Additional file 1: Figure S1).

### Functional annotation of unigenes

**Fig. 2:**
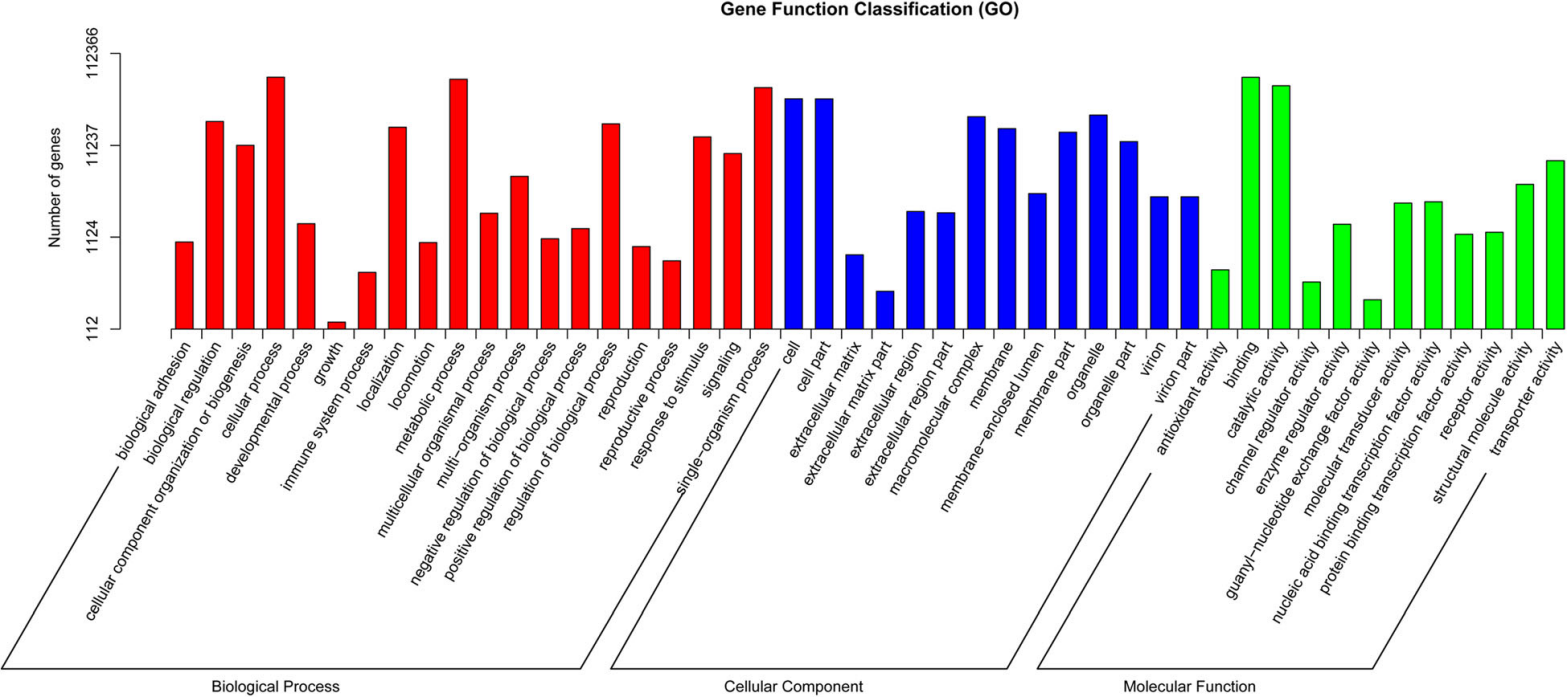
GO classifications of assembled unigenes. Unigenes were annotated in three main categories: biological process, cellular component and molecular function. The x-axis indicates the sub-categories and the y-axis indicates the number of unigenes

In order to obtain the comprehensive information of gene function, the unigenes were blasted using 7 databases (Nr, Nt, KEGG, SwissProt, PFAM, GO and KOG) (Table 1). In total 147,865 (55.84%) of all the assembled unigenes (264,791) could be annotated. Approximately 12,753 (4.81%) of the unigenes were simultaneously annotated by all databases. Nr analysis indicated that the top three species with the best hit were *Physcomitrella patens* (6.3%), *Selaginella moellendorffii* (5.3%) and *Guillardia theta* (4.5%), all belonging to original plants (Additional file 2: Figure S2). GO analysis was performed to classify the function of annotated unigenes and categorized 112,366 unigenes belonging to three categories (biological process, cellular component and molecular function) in which ‘cellular process’ (61,818), ‘cell’ (36,267) and ‘binding Molecular Function’ (61,638) were the primary groups, respectively (Fig. 2).

**Table 1:** Statistics of annotation analysis of unigenes

	Number of Unigenes	Percentage (%)	Software and Parameters
Annotated in Nr	100066	37.79	NCBI blast 2.2.28+, e-value = 1e-5
Annotated in Nt	31003	11.7	NCBI blast 2.2.28+, e-value = 1e-5
Annotated in KEGG	51831	19.57	KAAS, KEGG Automatic Annotation Server, e-value = 1e-10
Annotated in SwissProt	92286	34.85	NCBI blast 2.2.28+, e-value = 1e-5
Annotated in PFAM	110651	41.78	HMMER 3.0 package, hmmscan, e-value = 0.01
Annotated in GO	112366	42.43	Blast2GO v2.5 and self-write the Script, e-value = 1e-6
Annotated in KOG	67950	25.66	NCBI blast 2.2.28+, e-value = 1e-3
Annotated in all Databases	12753	4.81	–
Annotated in at least one Database	147865	55.84	–
Total Unigenes	264791	100	–

**Fig. 3:**
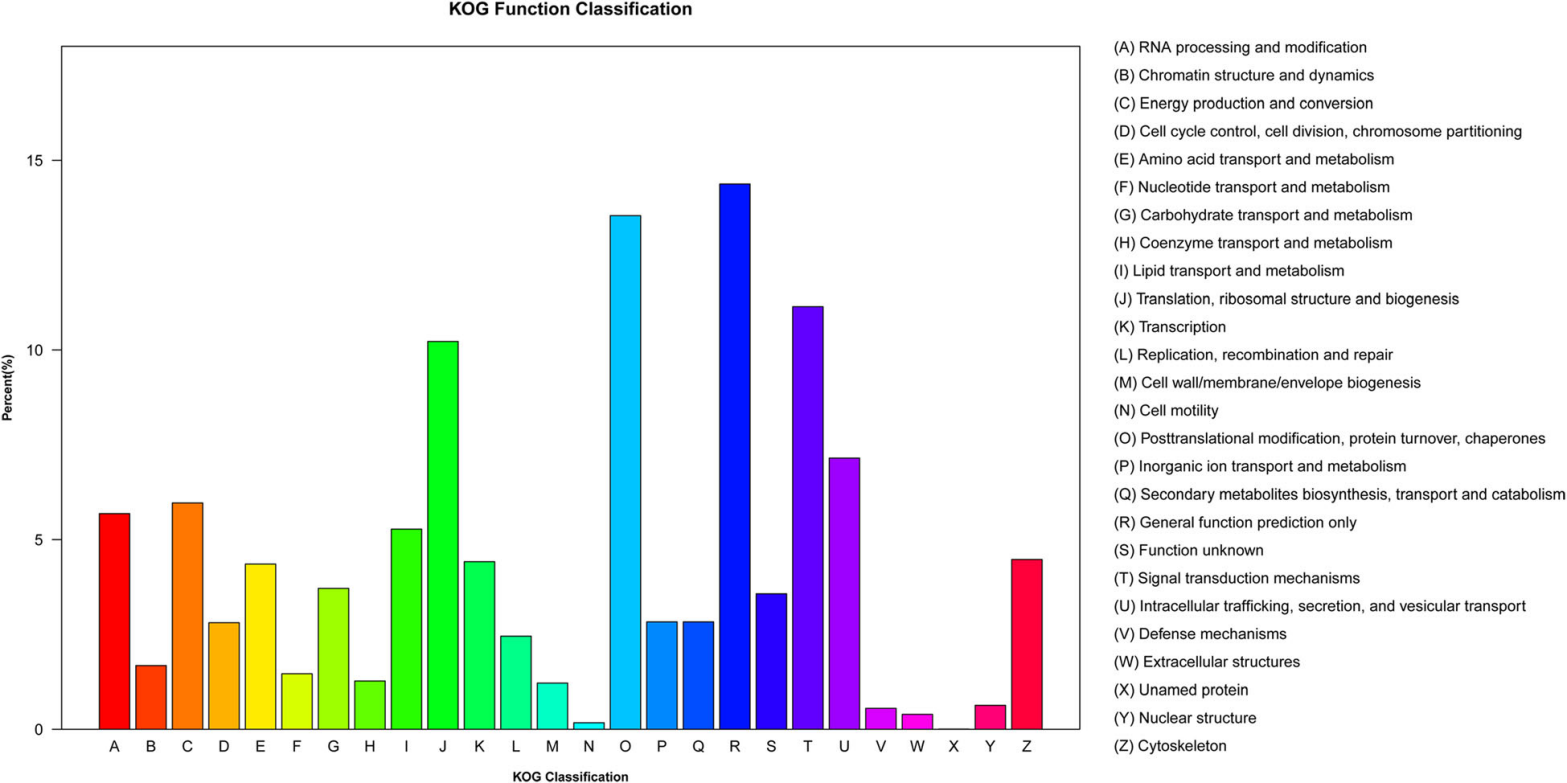
KOG classifications of assembled unigenes. Out of 264,791 de novo assembled unigenes, 67,950 were annotated and separated into 26 categories

To further evaluate the annotated transcriptome data, the sequences were compared with the KOG database. As a result, 67,950 unigenes were grouped into 26 categories (Fig. 3). ‘General function prediction only’ was the topmost category, with 9767 unigenes (14.37%), followed by ‘Posttranslational modification, protein turnover, chaperones’ (9199, 13.54%), ‘Signal transduction mechanisms’ (7572, 11.14%), ‘Translation, ribosomal structure and biogenesis’ (6948, 10.23%), ‘Intracellular trafficking, secretion, and vesicular transport’ (4854, 7.14%), ‘Energy production and conversion’ (4056, 5.97%), and ‘RNA processing and modification’ (3864, 5.69%). Furthermore, 2426 (3.57%) unknown functional unigenes were identified.

**Fig. 4:**
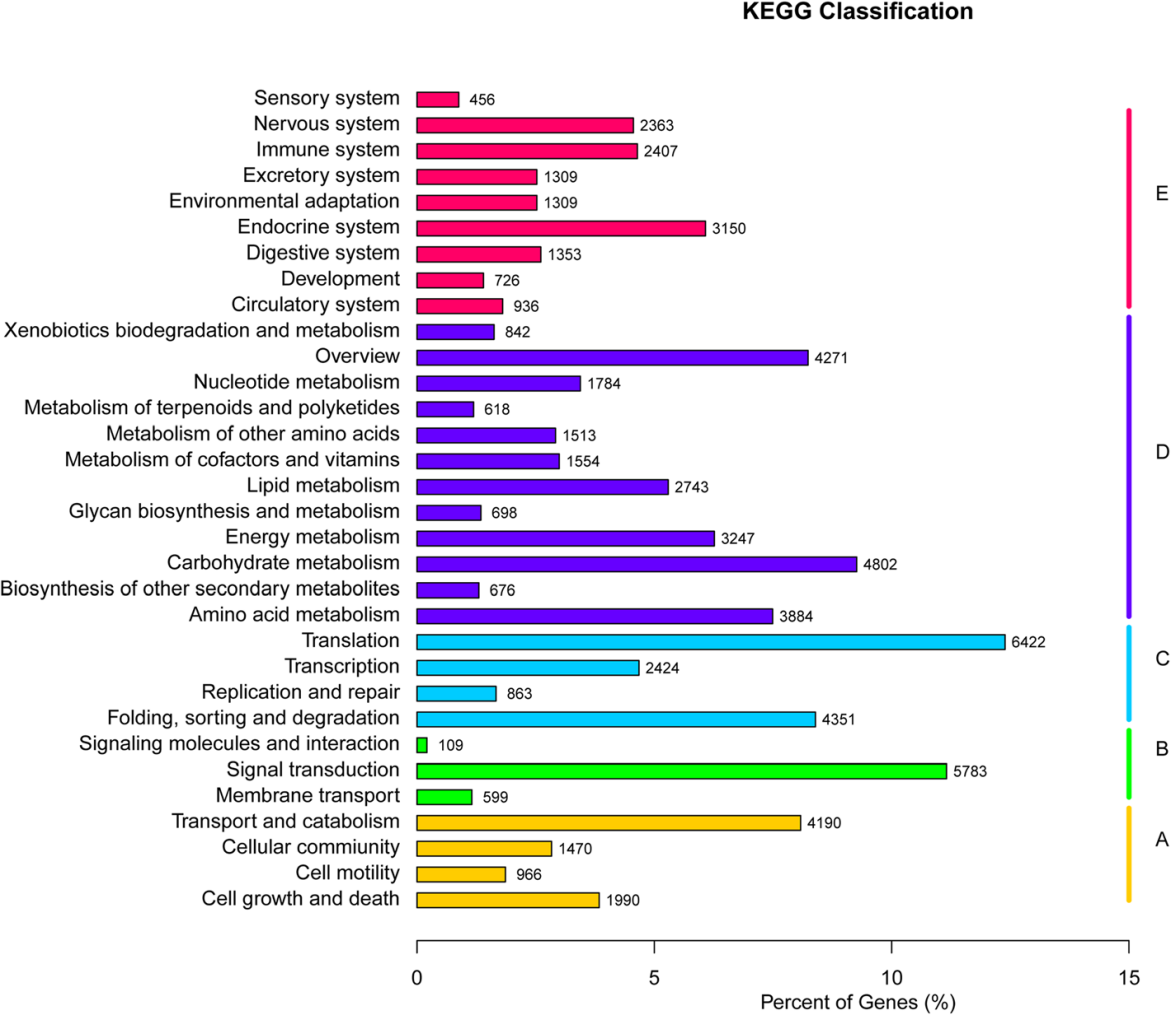
Distribution of the number of genes expressed in various metabolic pathways. **a** Cellular processes; **b** Environmental information processing; **c** Genetic information processing; **d** Metabolism; **e** Organismal systems

In addition, 51,831 unigenes were clustered to 32 KEGG pathways (Fig. 4). The majority of unigenes were grouped into pathways for ‘Translation’ (6422), ‘Signal transduction’ (5783), ‘Carbohydrate metabolism’ (4802) and ‘Overview’ (4271).

### Differential expressed genes (DEGs) analysis

**Fig. 5:**
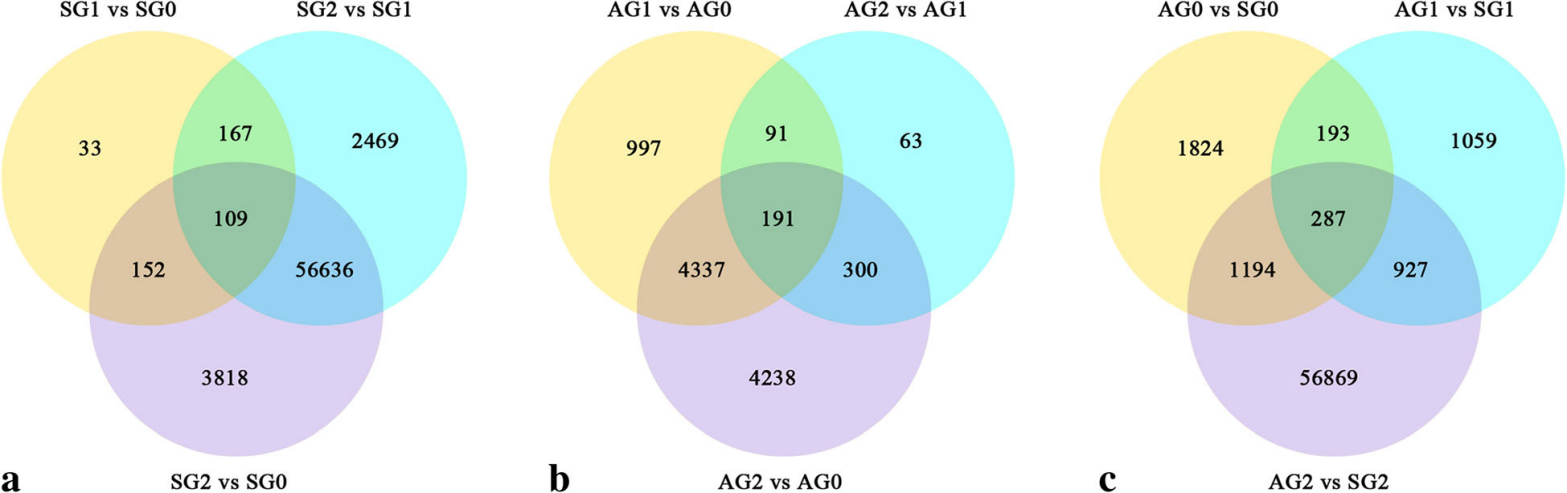
The pairwise comparisons of six stages. **a** Venn diagram of DEGs in sexual reproduction at three stages; **b** Venn diagram of DEGs in apogamy at three stages; **c** Venn diagram of DEGs between two reproductive modes at the corresponding stages

Differences in gene expression for six stages between the two reproductive processes were characterized, and DEGs were identified by pairwise comparisons across the six stages (Fig. 5). In sexual reproduction, 461, 59381 and 60715 DEGs were identified in pairs of SG1 vs SG0, SG2 vs SG1 and SG2 vs SG0 stages respectively. In apogamy, 5616, 645 and 9066 DEGs were identified corresponding to AG1 vs AG0, AG2 vs AG1 and AG2 vs AG0 stages. The total number of DEGs was higher in sexual reproduction compared with apogamy (Fig. 5a, b). In apogamy, the number of DEGs identified in early period (AG0 vs AG1, 5616) was more than that in later period (AG1 vs AG2, 645). On the other hand, this situation was reverse in sexual reproduction. The number of DEGs between SG0 and SG1 (461) was much fewer than that between SG1 and SG2 (59,381). Furthermore, the number of DEGs between the corresponding stages of the two reproductive modes exceeded comparisons between different stages in each reproductive mode (Fig. 5c).

### Validation of RNA-seq data by quantitative real-time PCR (qPCR) analysis

**Fig. 6:**
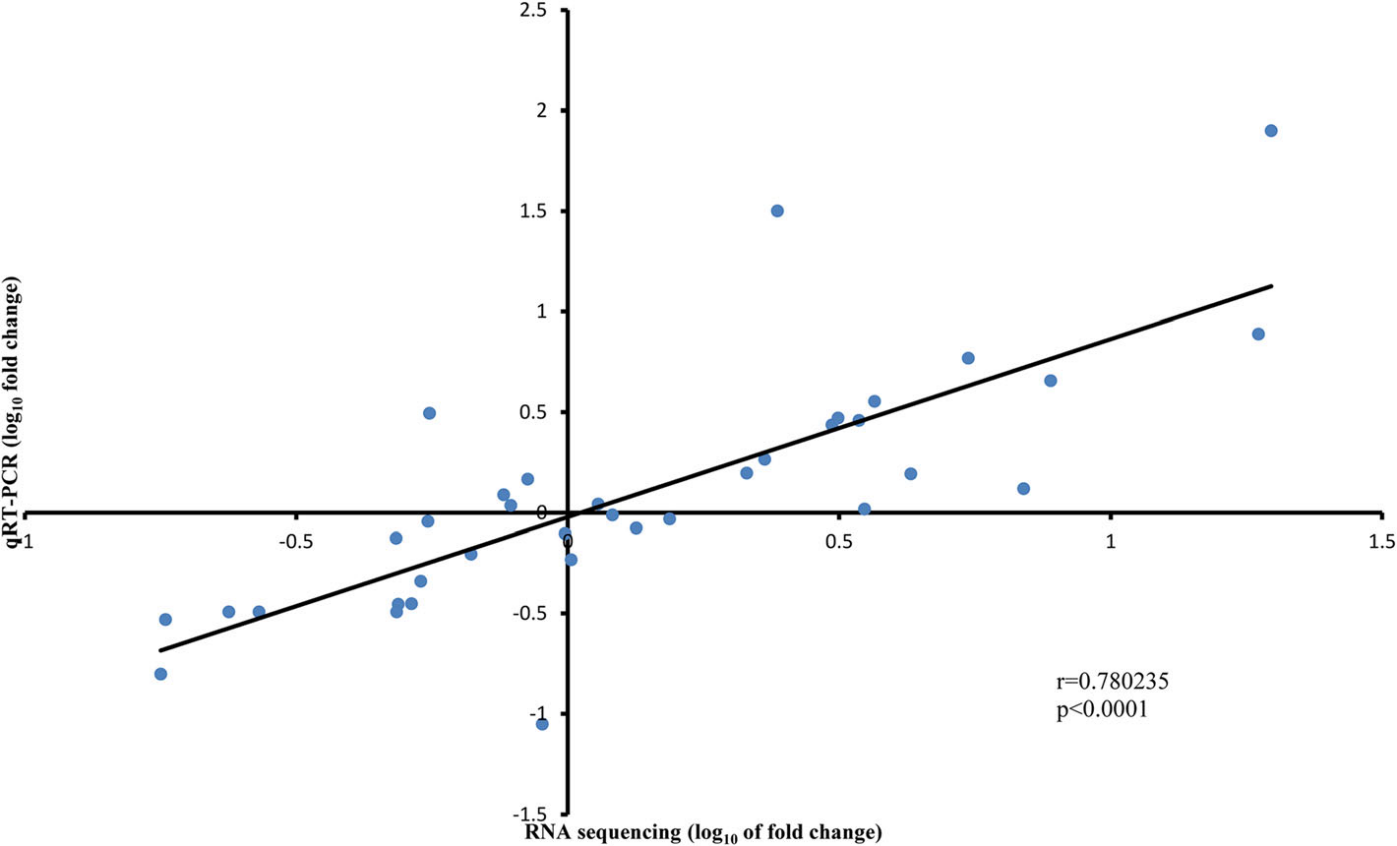
Correlation between qPCR and RNA sequencing for the nine selected genes. Each point represents a value of fold change of expression level at G1 or G2 comparing with that at G0 or G1. Fold-change values were log_10_ transformed

To confirm the reliability of the transcriptome analysis in the present study, nine DEGs were selected randomly and their expression levels were validated by qPCR. The fold change determined by RNA-seq and qPCR showed a good consistency (r = 0.780235, *p* < 0.0001), demonstrating that the transcriptome analysis was accurate (Fig. 6). In addition, the expression levels of qPCR were consistent with the results of RNA-seq for each gene (Additional file 3: Figure S3).

### KEGG pathway classification of DEGs during sexual organ and asexual embryonic cell initiation

To understand the biological functions of all the DEGs during sexual organ initiation, KEGG pathway enrichment analysis was carried out between SG0 and SG1. In total, 112 unigenes were clustered to 54 KEGG pathways, among which, only 10 pathways were significantly enriched (FDR ≤ 0.05) (Additional file 4: Table S1, Additional file 5: Figure S4, a), including circadian rhythm, estrogen signaling and protein processing in endoplasmic reticulum pathway. Similarly, 2287 unigenes were clustered to 293 KEGG pathways, in which, 37 pathways were significantly enriched (FDR ≤ 0.05) (Additional file 6: Table S2, Additional file 5: Figure S4, b) between AG0 and AG1, including flavone and flavonol biosynthesis, flavonoid biosynthesis, plant hormone signal transduction and starch and sucrose metabolism. The results of pathway enrichment analysis (Additional file 4: Table S1, Additional file 6: Table S2) contained pathways related to disease such as Toxoplasmosis, Measles, Influenza A and Pertussis. The genes involved in these pathways might be related to regulate the synthesis of compounds curing the disease in the fern. The pathways of the two reproductive modes that the DEGs enriched in showed some differences. Considering the role of carbohydrates metabolism and plant hormones in inducing apogamy, starch and sucrose metabolism and plant hormone signal transduction pathways that only significantly enriched in AG0 vs AG1 were first concerned.

### Genes in starch and sucrose metabolism pathway related to asexual embryonic cell initiation

**Fig. 7:**
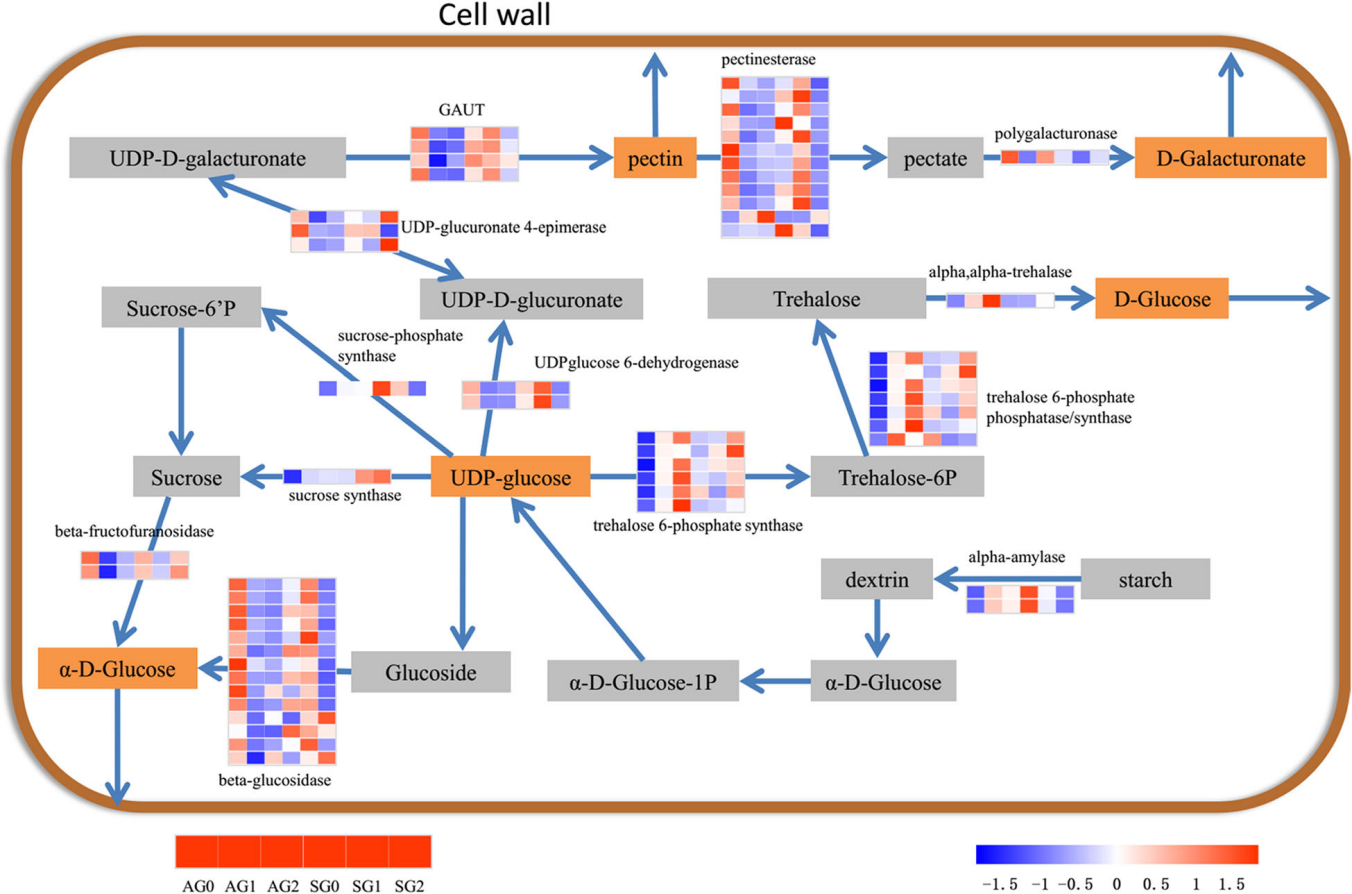
Transcriptional changes of genes involved in starch and sucrose metabolism pathway in the initial stages

KEGG pathway analysis presented that starch and sucrose metabolism pathway was only significantly enriched in asexual embryonic cell initiation. In total 57 unigenes were derived from identified DEGs involved in starch and sucrose metabolism pathways in Additional file 6. The expression levels of these genes were compared between the two reproductive patterns (Fig. 7, Additional file 7: Table S3, Additional file 8: Data S1). Most of the genes involved in glucose and pectin metabolism pathway, including beta-glucosidase, pectinesterase and UDP-glucuronate 4-epimerase, were down-regulated at AG1 stage compared with AG0 stage, and most of them had higher expression in SG1 stage. Sucrose, amylum and trehalose related genes, such as sucrose-phosphate synthase (*SPS*), alpha-amylase and trehalose 6-phosphate synthase (*TPS*) were up-regulated at AG1 stage compared with AG0 stage, while showed reverse trends between SG1 stage and SG0 stage.

### Genes in the plant hormone signal transduction pathway

**Fig. 8:**
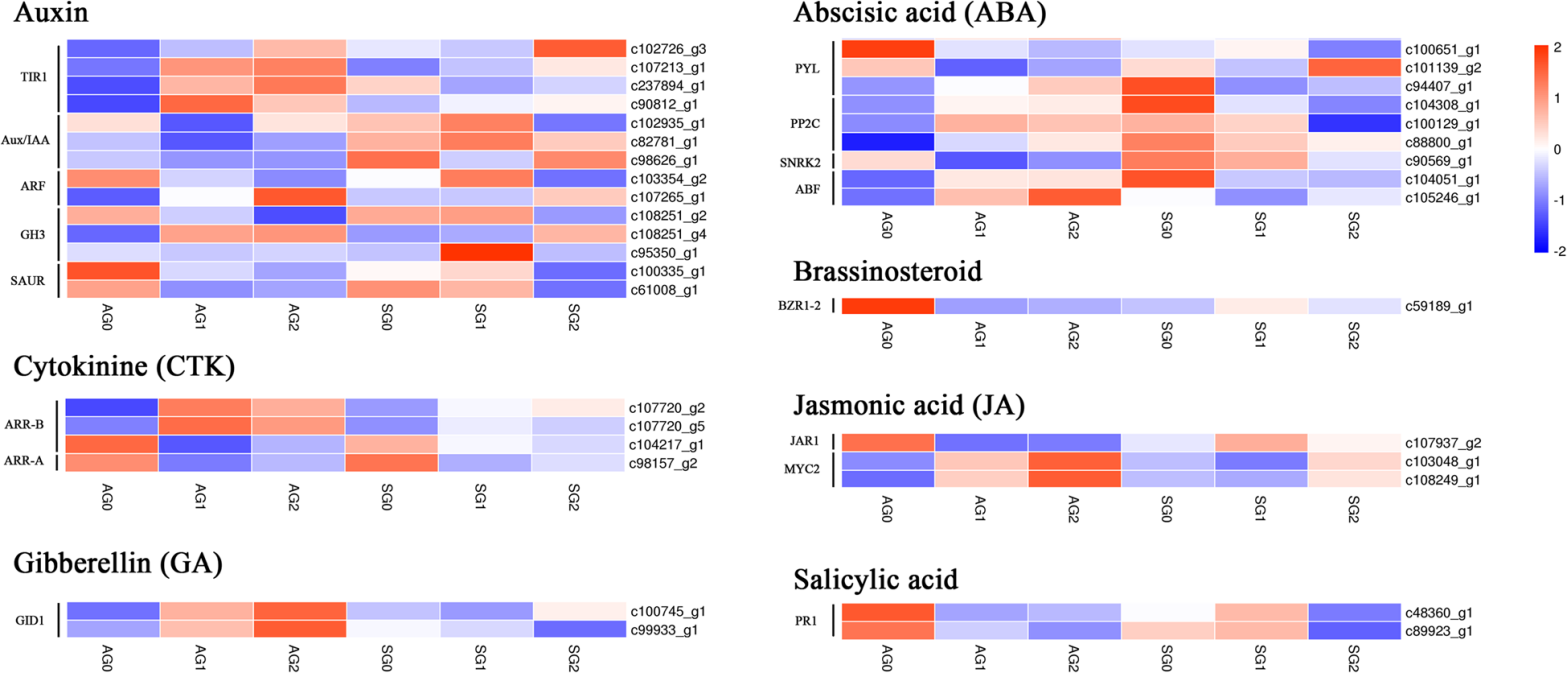
Transcriptional changes of genes associated with plant hormone signal transduction pathway in gametophytes

The regulation of exogenous hormones plays a key role in induction of apogamy in the ferns. According to the transcriptome data, plant hormone signal transduction pathway was enriched significantly accompanied with the differentiation of asexual embryonic cells (Additional file 6: Table S2), and appeared clearly in the top-20 enriched KEGG pathways during the sexual organ differentiation process (Additional file 4: Table S1). The expression levels of 35 genes (33 from Additional file 6, Table S2 and 2 from Additional file 4, Table S1) were compared at the six stages between the two reproductive patterns (Fig. 8, Additional file 9: Table S4, Additional file 10: Data S2).

In auxin signal transduction pathway, four transport inhibitor response protein 1 (*TIR1*) genes (c102726_g3, c107213_g1, c237894_g1, c90812_g1) were identified to express increasingly accompanied with the differentiation of asexual embryonic cells, while the expressions had no obvious changes during the sexual organ differentiation. Three *Aux/IAA* genes were down-regulated at AG1 stage compared with AG0 stage. Interestingly, one *GH3* gene (c95350_g1) was expressed extremely at higher level at SG1 than any other stages.

Two gibberellin insensitive dwarf1 (*GID1*) genes (c100745_g1, c99933_g1) associated with gibberellin metabolism showed higher expression levels in AG1 than in AG0, while the expression levels decreased during sexual organ differentiation.

In abscisic acid metabolism, most genes were up-regulated significantly between AG1 and AG0, while were down-regulated between SG1 and SG0, such as protein phosphatase 2C (*PP2C*) (c104308_g1, c100129_g1, c88800_g1) and ABRE binding factors (*ABF*) (c104051_g1, c105246_g1).

*MYC2*, a *bHLH* transcription factor, plays a crucial role in the jasmonic acid signalling pathway. Two *A. reniforme* var. *sinense* genes were identified: c103048_g1 and c108249_g1. Both were highly expressed at AG1 stage compared with AG0 stage while showed lower expression at SG1 than SG0. However, the *JAR1* (c107937_g2) gene showed reverse expression trend.

In salicylic acid phytohormone pathway, two *PR1* genes were identified: c48360_g1 and c89923_g1, both genes were down-regulated with the differentiation of asexual embryonic cells and showed higher expression levels at SG1 stage.

## Discussion

### RNA sequencing and de novo assembly of gametophyte transcriptome

Revealing the regulatory mechanisms of apogamy by RNA-seq provides an advantageous resource for investigations into the genetic control of apogamy in ferns and comparisons with the asexual processes of higher plants. In *C. richardii*, a SSH study was conducted and 170 unique sequences associated with apogamy commitment were detected [13]. In this study, many more unigenes and pathways responsible for apogamy were identified using RNA-seq technology, which may thus be helpful to further explain the mechanism inducing apogamy and sexual reproduction at transcriptional level.

In total, 147,865 unigenes were annotated to the 7 databases, which rated 55.84% of all the assembled unigenes (264,791). The abundant information of gene number and annotation was more than other studies using next-generation sequencing techniques to characterize the transcriptome of fern gametophytes, such as *Pteridium aquilinum* and *Lygodium japonicum* [21, 22].

### Global changes of gene expression between sexual reproduction and apogamy

Compared to sexual reproduction, less DEGs were identified in apogamy as a whole. By pairwise comparisons, the number of DEGs between AG0 and AG1 was more than that between AG1 and AG2, indicating that more complicated behaviors at transcriptional level occurred in asexual embryonic cell differentiation. However, the situation was inverse in sexual reproduction. The sporophyte formation phase had much more DEGs than sexual organ differentiation phase. These results suggested that the two reproductive patterns had different transcriptional regulatory networks. In addition, the factors affecting the initiation of apogamy had clearly developed a more complicated mechanism.

### Cell wall biosynthesis should be one of the key processes affecting the apogamy initiation in the ferns

In the current study, apogamy initiation affected by the starch and sucrose metabolism pathway was investigated. Through the comparison of KEGG enrichment analysis, the sexual organs and asexual embryonic cells differentiation had different metabolic pathways and transcriptional profiles. The starch and sucrose metabolism pathway related to cell wall biosynthesis was only significantly enriched in the stage of asexual embryo differentiation. The DEGs involved in this pathway were expressed diversely in apogamy and sexual reproduction. The genes related to *TPS/TPP*, amylase, sucrose synthase (*SUS*) and *SPS* all expressed actively while the genes related to beta-glucosidase and pectinesterase were suppressed during the process.

The amylase, *SUS* and *SPS* are the plant enzymes thought to play a positive role in cell wall synthesis [23–26], while the beta-glucosidase and pectinesterase are considered to degrade the cell wall [27, 28]. The change of expression levels of related genes in this pathway exactly demonstrated the cell wall was facilitated to generate during the asexual embryo differentiation process. Early apogamy involves differentiated somatic cells obtaining embryogenic competence and proliferating as embryogenic cells. This demands strict spatio and temporal control over cell division and elongation. The initiation of cell division is a key event during initial as well as subsequent steps of apogamy. Thus, apogamy should rely on accumulation of cell wall and regulation of signal molecules connected with the cell wall. The expression of genes mentioned above that responsible for cell wall might lead to the initiation of apogamy. However, it is necessary to validate the regulating relationship between the genes and apogamy experimentally in the future.

Moreover, the expression of *SPS* related gene (c108270_g1) was up-regulated and down-regulated during the differentiation of asexual embryo and sexual organ respectively. The expression of *SUS* (c105466_g1) maintained up-regulated in both processes. The expression trends of the genes were similar in the proliferation of somatic embryos in Picea. The activities of *SPS* increased while *SUS* levels stayed constant during the proliferation of somatic embryos [29, 30]. *SUS* and *SPS* are the plant enzymes believed to play a major role in sucrose biosynthesis and cellulose synthesis for cell wall [23–25]. The effects of sucrose on induction, maintenance, and maturation of somatic embryos also have been studied [31]. Taken together, the results showed that the *SPS* related genes might participate in regulation of asexual embryonic cells induction in apogamy.

In the present study, the *TPS/TPP* related genes were up-regulated significantly during the asexual embryonic cells differentiation while were down-regulated or had no differential expression in the sexual organ differentiation. Notably, the *TPS/TPP* is regarded to modulate the plant development and verified to regulate cell shape and plant architecture [32, 33]. The cell shape is acquired during the process of cellular differentiation. Expression of *AtTPS1* has been involved in carbon metabolism and specific developmental phenotypes, such as altered shoot growth and transition to flowering [34]. In tobacco, expression of *TPS* resulted in lancet-shaped leaf morphology [35]. In Arabidopsis, *TPS* was demonstrated to involve in the control of pavement cell shape and trichome branching [32]. Referring to somatic cells of gametophytes were triggered into asexual embryonic cells and then changed the cell shape and gradually developed to be outgrowths (sporophytes), the morphological changes showed the *TPS* played a vital function in apogamy. All these results suggested that the trehalose metabolism pathway was more active in apogamy than sexual reproduction, and thus might be one of the key processes influencing the apogamy.

Furthermore, overexpression of poplar xylem sucrose synthase in tobacco leads to a thickened cell wall [36]. Homozygous null mutants of *AtTPS1* displayed cell wall thickening and altered morphology [37, 38]. In the present study, the appearance of the asexual embryonic cells differentiation regions on gametophytes turned dark green, indicating that the cell walls were thickened. It is inferred that the differential expression of the related genes might participate in the process and explained why the color of the asexual embryonic cells was deepened.

### Regulation of plant hormone related genes was more active in apogamy initiation

In many fern species, apogamy can be effectively induced in suitable media supplemented with various plant hormones [10, 39] and phytohormone signaling proteins were supposed to participate in the apogamy [15]. It was noted in this study that more plant hormone related genes were regulated significantly during the early stage of apogamy, suggesting the embryogenic transition might be controlled by the expression of these genes.

The cell division is an important phenomenon for somatic cells dedifferentiation in apogamy. The auxin regulates cell division through causing hyperpolarization of the plasma membrane [40]. This is also a precondition for various auxin-triggered biological actions [41]. Auxin signaling is considered to play an important role in the molecular mechanism that controls the embryogenic transition of plant somatic cells [42]. In seed plants, auxin is one of the efficient initiators of somatic embryogenesis [43, 44]. In the present study, the comparative KEGG enrichment analysis between the two reproductive patterns in the fern indicated that numerous auxin-related genes, such as *TIR1*, *Aux/IAA*, *ARF*, *GH3* and *SAUR*, were transcribed significantly during the apogamy initiation. As the auxin receptor, the expression levels of *TIR1* genes were all up-regulated during early apogamy, indicating that auxin-signaling pathway functioned actively. The expression of *ARF1* gene (c103354_g2) gradually declining in apogamy was similar to the gene expression in somatic embryogenesis-induced explants in Arabidopsis [42]. These results indicated that auxin-related genes in the study should be critical regulators in influencing vegetative-to-embryogenic transition in apogamy initiation.

On the other hand, the *GH3* gene (c95350_g1) involved in the auxin pathway enriched in the sexual organ initiation had dramatically higher expression at SG1 stage than other stages. In soybean, *GH3* expression is restricted largely to specific tissues within organs of the developing flowers and expressed transiently during flower development [45]. This demonstrated that this gene might transiently participate in the sexual organ initiation.

Gibberellins are plant hormones directly related to important plant growth and development events including flowering and ovule development [46, 47]. *GID1* is a gibberellin receptor previously identified in plants and associated with reproductive development [48]. In the present study, two *GID1* genes were induced in the two reproductive patterns. This result was similar to the findings in Arabidopsis where the expression of *GID1* was essential for reproduction [48, 49]. The expression levels of *GID1* increased and decreased during the initial stage of apogamy and sexual reproduction respectively, indicated that contrary effects of gibberellin regulation between the two reproductive patterns. In *Brachiaria brizantha*, *GID1* is only expressed in the nucellus of apomixes compared with sexual reproduction, previous to aposporous initial differentiation [50]. There are sufficient reasons to believe that the function of *GID1* might be related to apogamy initiation and conserved in evolution.

Plant hormone ABA is discovered in a wide range of land plants, from mosses to angiosperms. The ABA signaling is associated with both the abiotic stress responses and developmental regulation [51, 52]. The genes, c104308_g1, c100129_g1 and c88800_g1, were annotated as *PP2Cs*, the abundances of which increased significantly from AG0 to AG1 and decreased from SG0 to SG1. Interestingly, the *PP2Cs* were demonstrated to alter morphogenesis in Physcomitrella [53]. The transgenic plants overexpressing *PP2Cs* had continuous growth of archegonia (female organ) yet with few sporophytes forming compared to the wild type. Considering no fertilization occurs in the apogamy, these results indicated that the *PP2Cs* might have the effect of inhibiting the function of sexual organs.

Signal transduction pathways were also analyzed for other hormones, jasmonic acid and salicylic acid, according to the KEGG enrichment analysis. The genes, having increasing and decreasing expression levels in apogamy and sexual reproduction respectively, including *ABF*, *MYC2* related to asexual embryonic initiation were observed. Notably, these genes have been proved to play an important role in stress responses [54, 55]. It has been suggested that an increase in metabolic activity and stress responses together induces the apogamy commitment in *C. richardii* [13] and many proteins related to responses to biotic or abiotic stimuli are identified in the apogamous fern *Dryopteris affinis* ssp. *affinis* [15]. Accordingly, it is speculated that apogamy might be adapted to a harsh environment in early evolution.

Generally, plant biological activity is not only modulated by one hormone signal transduction pathway, thus the biological phenomenon often represents the results of the combined interplay of several different hormone signal transduction pathways. The interactions of these hormone-responsive genes for affecting apogamy need to be verified in future.

## Methods

### Plant material

The spores of *A. reniforme* var. *sinense* were obtained from mature sporophylls of the sporophytes cultured in the greenhouse at Huazhong Agricultural University (Wuhan, China). The gametophytes were induced from the spores and sterile cultured in the culture room, in Laboratory of Genetic Resources of Landscape Plants, Huazhong Agricultural University (Wuhan, China). The culture condition was under a 16/8-h (light/dark) photocycle at 25/20 °C (day/night).

Two reproductive modes of gametophytes, sexual reproductive gametophytes (SG) and apogamous gametophytes (AG), were used for transcriptome analysis. Total six stages of tissues from two reproductive modes were collected. The sexual reproductive gametophytes contained: gametophytes with no sexual organs differentiation (SG0), gametophytes with sexual organ initiation (SG1), gametophytes with the sporophytes appearing (SG2). The apogamous gametophytes contained: gametophytes with no agamous buds appearing (AG0), gametophytes with agamous embryonic cells initiation (AG1), gametophytes with the apogamous sporophytes appearing (AG2). The samples of each stage were sampled at the same time, transferred immediately to liquid nitrogen, and stored subsequently at − 80 °C until RNA extraction. Three biological replicates were collected for each stage (except the SG2 stage had two replicates). These samples were also collected and frozen immediately in liquid nitrogen and kept at − 80 °C for the experiments of qPCR analysis.

### RNA isolation, library construction and sequencing

Total RNA was extracted using the RNeasy Mini Kit (Qiagen, Valencia, CA, USA) according to the protocol provided by manufacturer. The integrity of RNA was determined by electrophoresis in 1% agarose gel. RNA quantity and quality were assessed using a NanoDrop™ 2000 spectrophotometer (Thermo Scientific) and an Agilent 2100 Bioanalyzer (Agilent Technologies, CA, USA). The samples showing A260/A280 ratio of 1.8–2.0 and an A260/A230 ratio of 2.0–2.2, and with RNA integrity number (RIN) greater than 8.0 were used for subsequent analysis. The samples were subsequently used in cDNA library construction and Illumina sequencing which was completed by Beijing Novogene Bioinformatics Technology Co., Ltd.

A total amount of 3 μg RNA per sample was used as input material for the RNA sample preparations. Sequencing libraries were generated using NEBNext® Ultra™ RNA Library Prep Kit for Illumina® (NEB, USA) following manufacturer’s recommendations and index codes were added to attribute sequences to each sample. Briefly, mRNA was purified from total RNA using poly-T oligo-attached magnetic beads. Fragmentation was carried out using divalent cations under elevated temperature in NEBNext First Strand Synthesis Reaction Buffer (5X). First strand cDNA was synthesized using random hexamer primer and M-MuLV Reverse Transcriptase (RNase H^−^). Second strand cDNA synthesis was subsequently performed using DNA Polymerase I and RNase H. Remaining overhangs were converted into blunt ends via exonuclease/polymerase activities. After adenylation of 3′ ends of DNA fragments, NEBNext Adaptor with hairpin loop structure were ligated to prepare for hybridization. In order to select cDNA fragments of preferentially 150~200 bp in length, the library fragments were purified with AMPure XP system (Beckman Coulter, Beverly, USA). Then 3 μl USER Enzyme (NEB, USA) was used with size-selected, adaptor-ligated cDNA at 37 °C for 15 min followed by 5 min at 95 °C before PCR. Then PCR was performed with Phusion High-Fidelity DNA polymerase, Universal PCR primers and Index (X) Primer. At last, PCR products were purified (AMPure XP system) and library quality was assessed on the Agilent Bioanalyzer 2100 system.

The clustering of the index-coded samples was performed on a cBot Cluster Generation System using TruSeq PE Cluster Kit v3-cBot-HS (Illumina) according to the manufacturer’s instructions. After cluster generation, the library preparations were sequenced on an Illumina Hiseq 2500 platform and 125 bp paired-end reads were generated.

The raw reads were cleaned by removing reads containing adapter, reads containing poly-N and low quality reads. High quality RNA-seq reads were pooled from Illumina sequencing of all samples and were then assembled into transcripts using Trinity (version 2.3.2) [56] with min-kmer-cov set to 2 by default and other parameters set at their defaults. After assembly, the longest transcripts of each gene were selected as the unigenes for subsequent analysis.

We quantified transcript levels in expected number of Fragments Per Kilobase of transcript sequence per Millions base pairs sequenced (FPKM) [57]. RSEM v1.3.0 was used to obtain FPKM values. RSEM used bowtie2 parameter mismatch 0, (bowtie2 default parameters).

### Annotation and differential gene expression analysis

The unigenes were annotated according to seven databases, NCBI blast (2.2.28+) was applied to search against the Nr database (E-value = 1 × 10^− 5^), Nt database (E-value = 1 × 10^− 5^), Swiss-Prot database (E-value = 1 × 10^− 5^) and COG/KOG database (E-value = 1 × 10^− 3^). Pfam annotation for the unigenes was finished using the HMMER 3.0 package [58], hmmscan (e-value = 0.01). The unigenes were annotated using GO database by Blast2GO v2.5 [59] with the self-write script (e-value = 1 × 10^− 6^) on the basis of annotation results of Nr and Pfam. For KEGG annotation [60], the KAAS and KEGG Automatic Annotation Server [61] (E-value = 1 × 10^− 10^) was conducted to confirm the metabolic pathways of unigenes.

Differential expression analysis of two stages was performed using the DESeq R package (1.10.1) [62]. DESeq provided statistical routines for determining differential expression in digital gene expression data using a model based on the negative binomial distribution. The resulting *p* values were adjusted using the q value method proposed by Storey et al. [63]. Genes with an adjusted p value < 0.05 and an absolute value of log_2_ fold-change > 1 found by DESeq were assigned as differentially expressed.

KEGG pathway enrichment analysis of the DEGs was done using KOBAS [61] with the hyper-geometric distribution model. For KEGG enrichment analysis, the pathway with a FDR value ≤0.05 was considered as an enriched pathway.

### Quantitative real-time PCR validation of RNA-seq

Nine genes with different expression patterns revealed by RNA sequencing were randomly selected for validation by qPCR. First strand cDNA was synthesized with TransSprit® One-Step gDNA Removal and cDNA Synthesis SuperMix (TransGen Biotech, China) according to manufacturer’s instructions. All cDNA were stored at − 20 °C until use. Specific primers for each gene were designed according to the gene CDS sequences using Primer 3.0 software (http://bioinfo.ut.ee/primer3-0.4.0/) and are listed in Table S5. The 40S ribosomal protein S9–2-like *(40S*) gene was used as internal control (Additional file 11: Table S5). The qPCR was performed on a 7500 Fast Real Time PCR system (Life Technologies, USA) using 2X SYBR qPCR Mix with low ROX concentration (Aidlab, China) according to the manufacturer’s instruction. Four technical replicates were adopted for each sample. For each gene, the full sample set was run on the same plate to exclude any technical variation. Relative transcription levels were analyzed using the 2^-∆∆Ct^ method. A regression analysis was performed between qPCR and RNA sequencing including all genes of the two reproductive modes at the six stages.

## Conclusions

In this study, it was concluded that different transcriptome regulating networks were developed between the sexual reproduction and apogamy in the fern. The genes regulating the apogamy initiation were more active than that in sexual reproduction. Then, the starch and sucrose metabolism relating to cell wall might play a key role in affecting apogamy initiation. The involved genes, such as *TPS/TPP*, *SUS* and *SPS* were regulated actively in this course. In addition, the hormone signal transduction pathway should play a prominent role in apogamy rather than sexual reproduction initiation. The candidate genes, including *TIR1*, *ARF1*, *GID1*, *PP2C*, *ABF* and *MYC2* were identified to participate in apogamy initiation. These results laid the foundation for revealing the molecular mechanisms of apogamy and would also be benefit for understanding the mechanisms related to asexual reproduction in higher plants. On the basis of the complicated apogamy and sexual reproduction transcriptomic changes in this study, further functional verification of the candidate genes and pathways would be needed in the future.

## Additional files

Additional file 1: **Figure S1.** Length distribution of transcripts and unigenes in the assembled transcriptomes. The x axis shows the lengths of transcripts/unigenes and the y axis shows the number of transcripts/unigenes. (TIF 292 kb)

Additional file 2: **Figure S2.** Analysis of the BLAST results in Nr database. (a) Similarity distribution; (b) E-value distribution; (c) Best hit species classification. (TIF 101 kb)

Additional file 3: **Figure S3.** qRT-PCR validation of differential gene expression for two reproductive modes of gametophytes in *Adiantum reniforme* var. *sinense*. (PDF 85 kb)

Additional file 4: **Table S1.** The significantly enriched pathways of all the DEGs between SG0 and SG1. (XLSX 16 kb)

Additional file 5: **Figure S4.** The top-20 enriched KEGG pathways. a, SG0 vs SG1; b, AG0 vs AG1. The Y-axis represents the pathway term; the X-axis represents the rich factor. The sizes of the points represent different DEG numbers, such that the bigger the point, the greater the DEG number. The colors represent different q-values. (TIF 730 kb)

Additional file 6: **Table S2.** The significantly enriched pathways of all the DEGs between AG0 and AG1. (XLSX 45 kb)

Additional file 7: **Table S3.** Expression levels of genes involved in starch and sucrose metabolism pathway in gametophytes. (XLS 34 kb)

Additional file 8: **Data S1.** Sequences of genes involved in starch and sucrose metabolism pathway in gametophytes. (TXT 113 kb)

Additional file 9: **Table S4.** Genes associated with plant hormone signal transduction pathway. (XLS 27 kb)

Additional file 10: **Data S2.** Sequences of genes associated with plant hormone signal transduction pathway. (TXT 74 kb)

Additional file 11: **Table S5.** Gene IDs, descriptions and primer sequences for the nine genes used for qRT-PCR verification. (XLS 28 kb)

## Data Availability

Raw data supporting our findings can be found in the National Center for Biotechnology Information (NCBI) database under the accession number PRJNA548821.

## Abbreviations

ABA: Abscisic acid
ABF: ABRE binding factors
DEGs: Differential expressed genes
GID1: GIBBERELLIN INSENSITIVE DWARF1
GO: Gene Ontology
KEGG: Kyoto Encyclopedia of Genes and Genomes
KOG: euKaryotic Ortholog Groups
Nr: NCBI non-redundant protein sequences
Nt: NCBI nucleotide sequences
PFAM: Protein family
PP2C: protein phosphatase 2C
qPCR: Quantitative real-time PCR
SPS: Sucrose-phosphate synthase
SUS: Sucrose synthase
TIR1: Transport inhibitor response protein 1
TPP: Trehalose 6-phosphate phosphatase
TPS: Trehalose 6-phosphate synthase
