# Supplementary figures and images for: Comparative transcriptome analysis of two reproductive modes in Adiantum reniforme var. sinense targeted to explore possible mechanism of apogamy
Source: BMC Genet. 2019 Jul 9;20:55. doi: 10.1186/s12863-019-0762-8 (PMC6617869; doi:10.1186/s12863-019-0762-8)

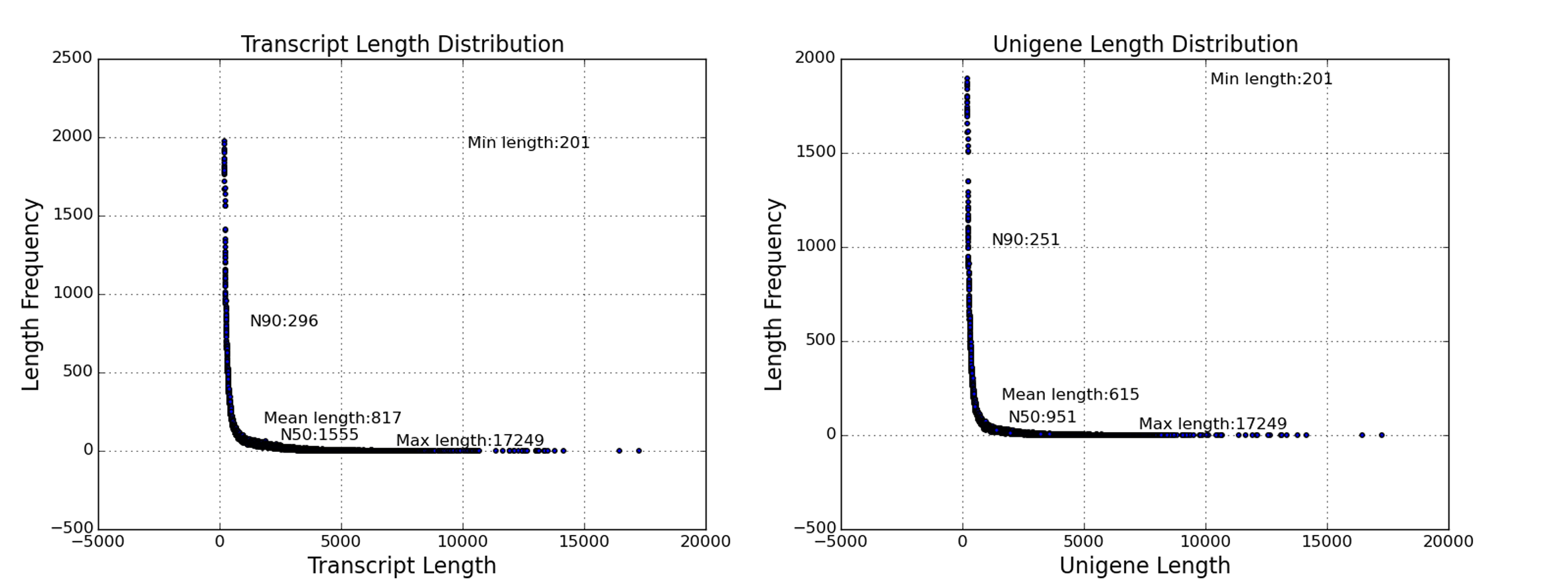

Supplement: Supplementary file 1 — Figure S1. Length distribution of transcripts and unigenes in the assembled transcriptomes. The x axis shows the lengths of transcripts/unigenes and the y axis shows the number of transcripts/unigenes. (TIF 292 kb) [file 12863_2019_762_MOESM1_ESM.tif]

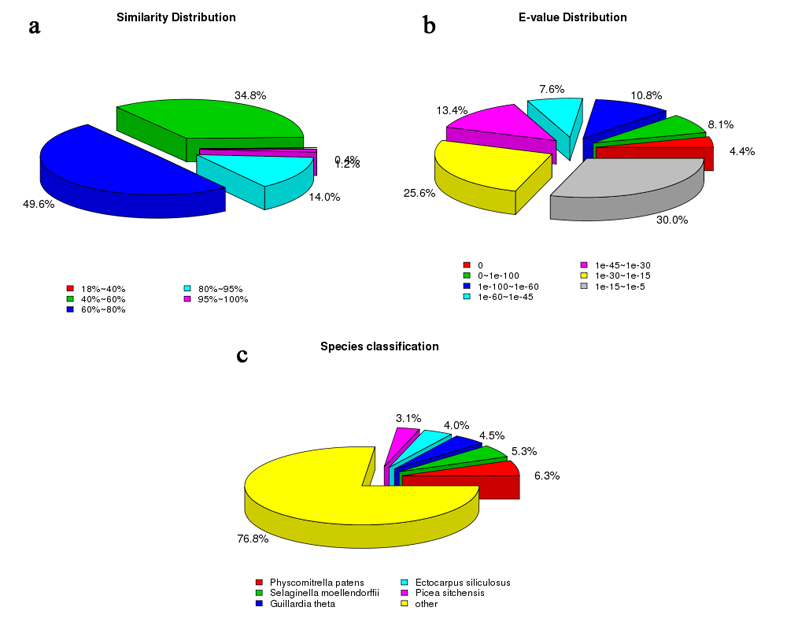

Supplement: Supplementary file 2 — Figure S2. Analysis of the BLAST results in Nr database. (a) Similarity distribution; (b) E-value distribution; (c) Best hit species classification. (TIF 101 kb) [file 12863_2019_762_MOESM2_ESM.tif]

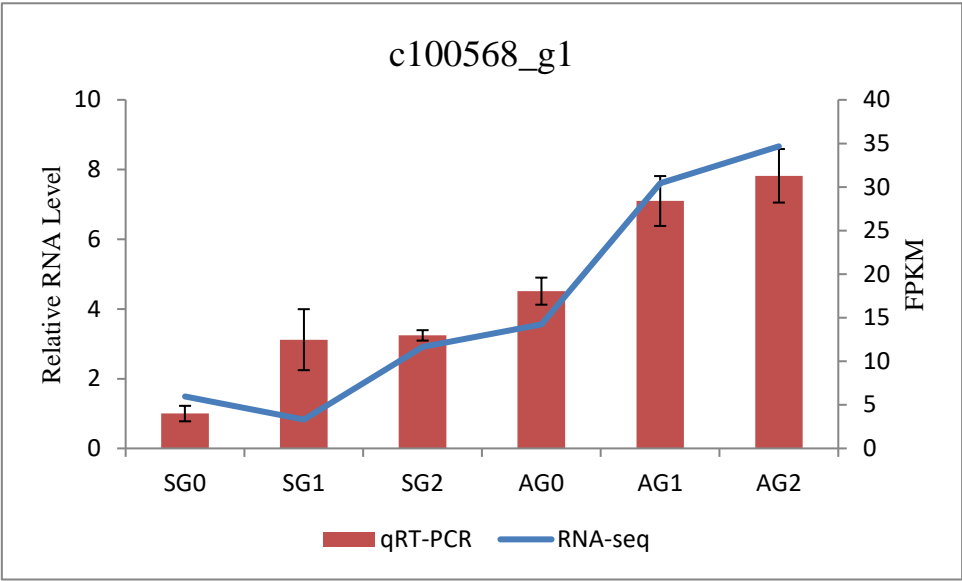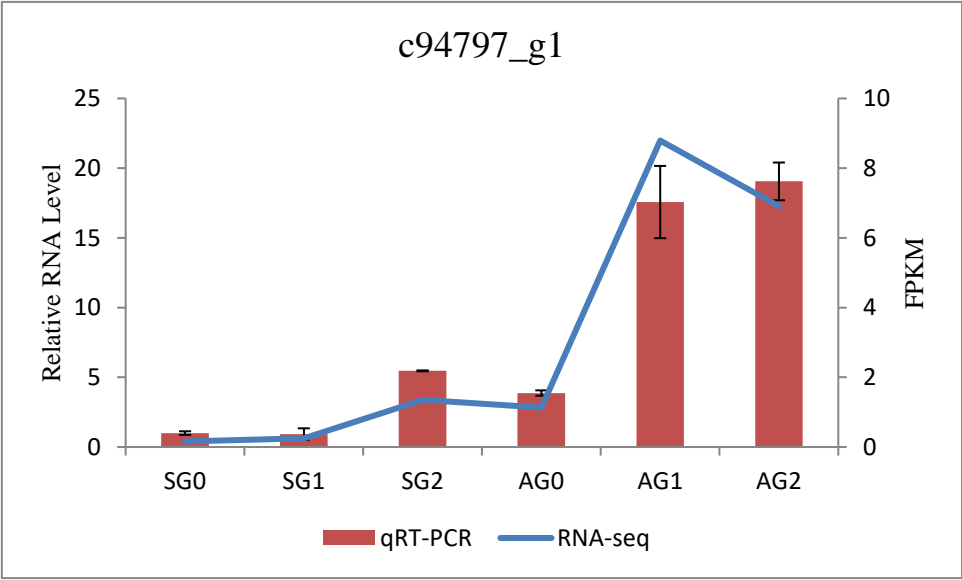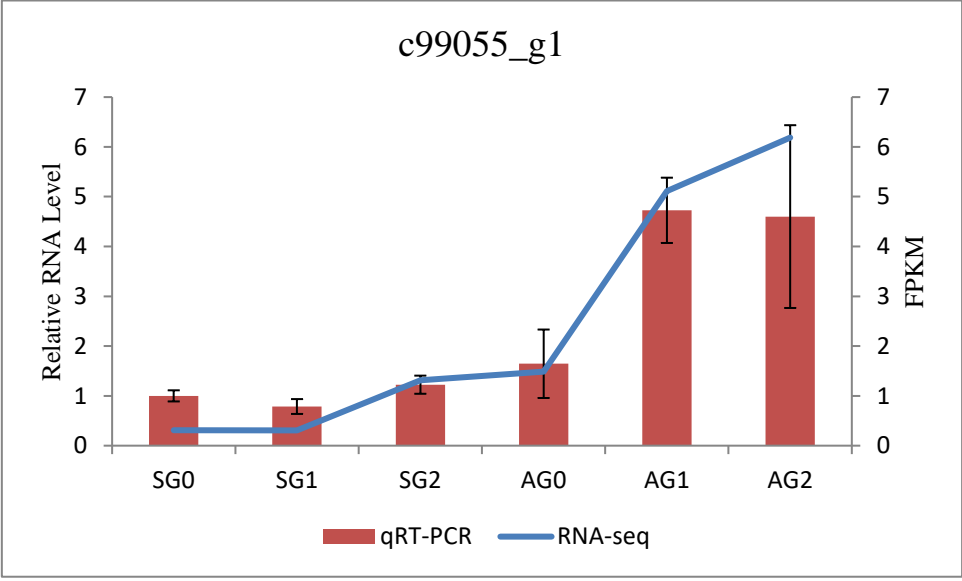

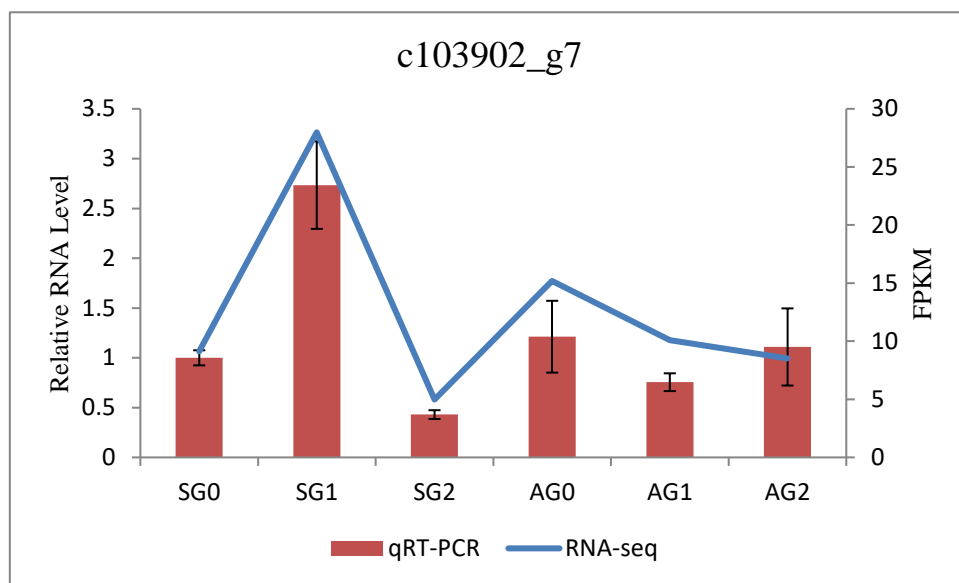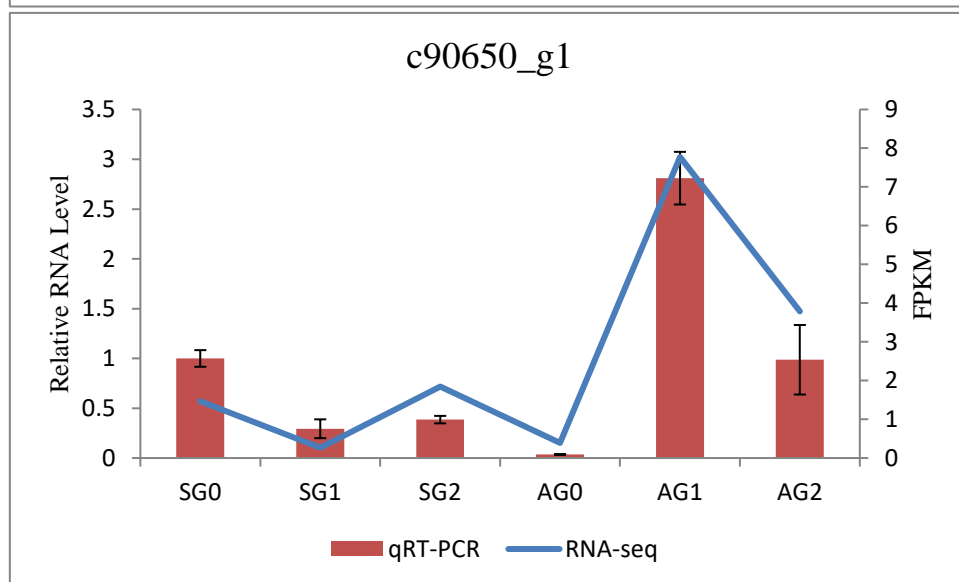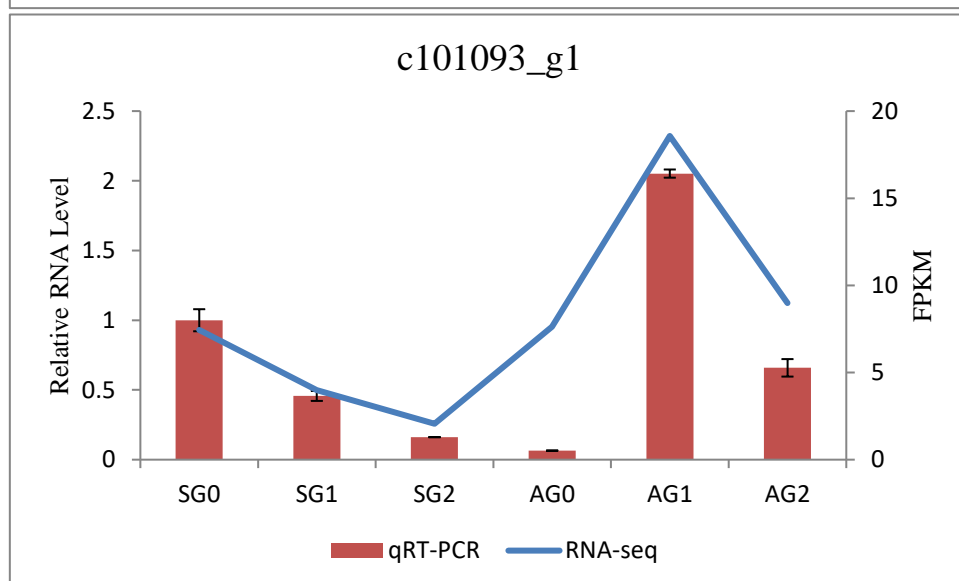

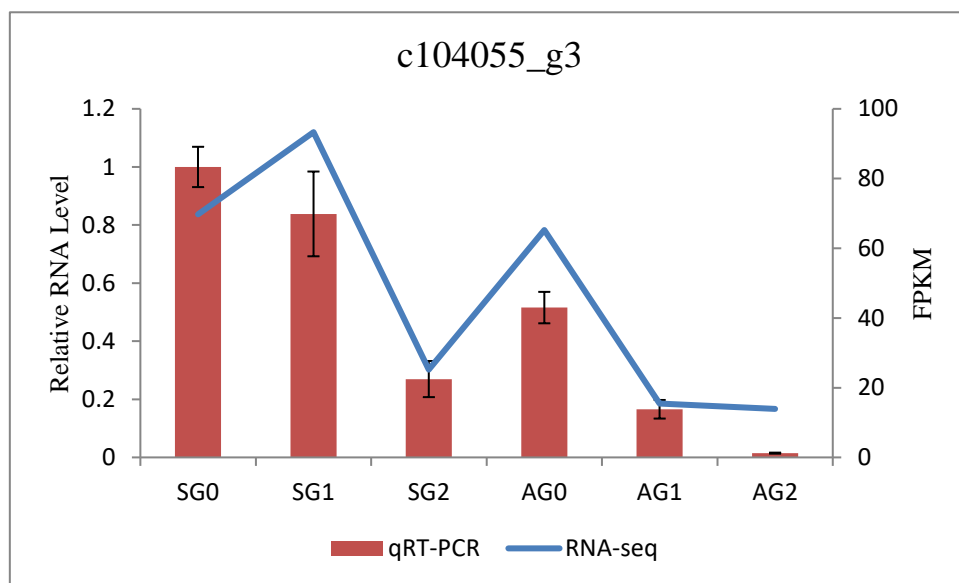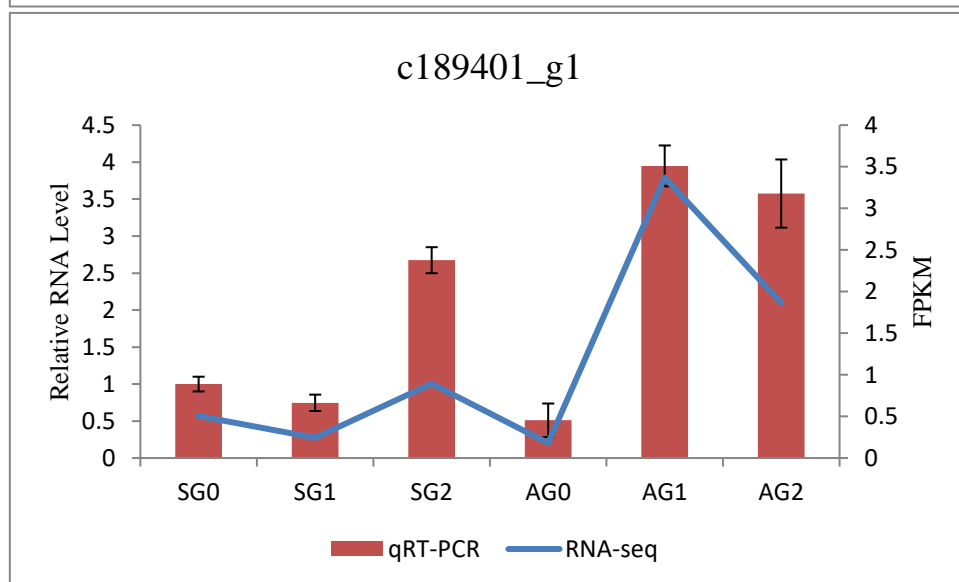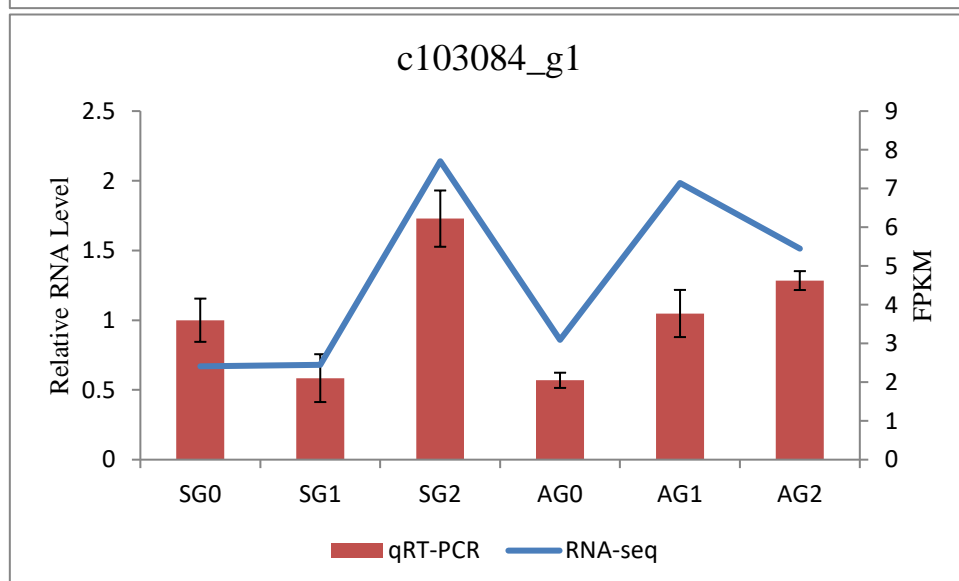

Supplement: Supplementary file 3 — Figure S3. qRT-PCR validation of differential gene expression for two reproductive modes of gametophytes in Adiantum reniforme var. sinense. (PDF 85 kb) [file 12863_2019_762_MOESM3_ESM.pdf]

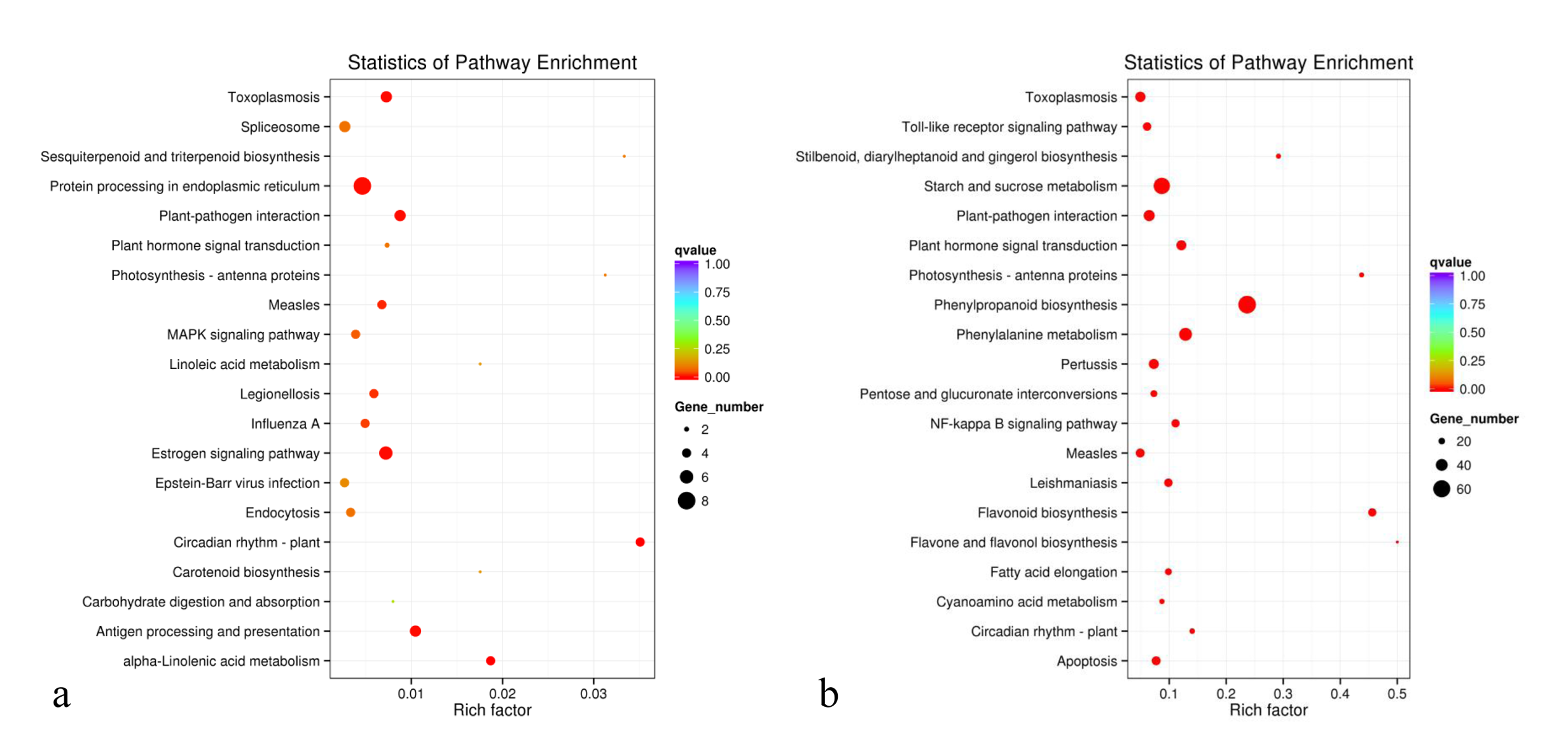

Supplement: Supplementary file 5 — Figure S4. The top-20 enriched KEGG pathways. a, SG0 vs SG1; b, AG0 vs AG1. The Y-axis represents the pathway term; the X-axis represents the rich factor. The sizes of the points represent different DEG numbers, such that the bigger the point, the greater the DEG number. The colors represent different q-values. (TIF 730 kb) [file 12863_2019_762_MOESM5_ESM.tif]
